# Exploring postoperative nasal airflow dynamics and impact on crust formation and mucosal healing after endoscopic tumor resection using computational fluid dynamics analysis

**DOI:** 10.1371/journal.pone.0323560

**Published:** 2025-05-12

**Authors:** Yang Na, Minhae Park, Yong Gi Jung

**Affiliations:** 1 Department of Mechanical Engineering, Konkuk University, Gwangjin-gu, Seoul, Republic of Korea; 2 Department of Otorhinolaryngology, Sanggye Paik Hospital, Inje University, School of Medicine, Seoul, Republic of Korea; 3 Department of Otorhinolaryngology-Head and Neck Surgery, Samsung Medical Center, Sungkyunkwan University, School of Medicine, Seoul, Republic of Korea; 4 Department of Data Convergence and Future Medicine, Sungkyunkwan University School of Medicine, Seoul, Republic of Korea; Macquarie University, AUSTRALIA

## Abstract

**Background:**

Postoperative observations in patients undergoing radical endoscopic resection surgery for sinonasal tumors have frequently noted the occurrence of mucosal crusts or bleeding on the nasal epithelial surface. This study employs computational fluid dynamics (CFD) techniques to elucidate the critical fluidic factors associated with these postoperative morbidities.

**Methods:**

Three-dimensional models of the postoperative nasal cavities were constructed using thin-section computed tomography data from nine patients who underwent radical resection surgery for sinonasal tumors, encompassing endoscopic medial maxillectomy (N = 3), endoscopic craniofacial resection (N = 3), and endoscopic resection with septectomy (N = 3). Simulations of inspiratory airflow, assuming turbulent flow, were conducted to analyze airflow and air conditioning characteristics at sites where crust formation occurred.

**Results:**

Frequent sites of crusting or bleeding were associated with the maxillary or sphenoidal sinuses (five out of nine subjects), where the ostia were substantially enlarged due to the surgery. Two distinct fluid dynamic features contributing to crust formation were identified. In five cases, crusts formed where local wall shear stress was elevated, while in four cases, crusts were observed in regions characterized by stagnant flow. Additionally, the relative humidity in the nasopharynx decreased to an unsatisfactory range (83.8‒85.7%).

**Conclusion:**

This study demonstrated that two distinct fluid dynamic environments conducive to postoperative crust formation are possible, indicating that the wall shear stress level alone is insufficient for crust formation. Additionally, impaired humidification function observed following the surgery underscores the necessity of providing adequate moisturization for post-surgical care.

## Introduction

The intricate regulation of airflow and conditioning of inhaled air within the nasal cavity is crucial for the homeostasis of the respiratory system [1–5]. However, surgical interventions for advanced paranasal sinus tumors often necessitate the removal of multiple intranasal structures, potentially disrupting natural airflow patterns and compromising nasal function [6–8]. Recent advancements in surgical techniques and multidisciplinary treatments, including radiation therapy and chemotherapy, have significantly improved treatment outcomes for sinonasal tumors. However, research on the alterations in nasal physiology due to inevitable changes in nasal cavity structures after treatment needs to be more comprehensive, and predicting postoperative functional outcomes remains challenging due to the complex dynamics of nasal airflow, an area where research is notably lacking [9,10].

With the growing interest in patients’ quality of life during treatment, there has been a greater recognition of postoperative functional problems, particularly due to substantial alterations in nasal airflow patterns. For instance, procedures primarily involving the resection of the inferior turbinate often result in empty nose syndrome (ENS), characterized by symptoms such as nasal obstruction, dryness, and disrupted sleep [11–13]. Similarly, multiple numerical studies have reported substantial changes in nasal airflow following endoscopic skull base surgery, which could potentially compromise nasal function [6,7,14]. These findings support the hypothesis that the extent and type of surgical technique play a critical role in postoperative morbidity, as observed in various clinical studies [10,15–18]. Notably, common sinonasal morbidities include nasal crusting and discharge [7,17], suggesting that postoperative morbidity may arise from the adverse consequences of altered airflow configurations. However, existing literature predominantly focuses on clinical outcomes and subjective symptoms, lacking quantitative assessments of airflow changes and their implications for air conditioning characteristics, with some exceptions [7,19].

This study aims to bridge this gap by analyzing nasal airflow patterns using Computational Fluid Dynamics (CFD) in patients who underwent three different types of endoscopic surgery for sinonasal tumors involving extensive resection of intranasal structure: endoscopic medial maxillectomy (EMM), endoscopic craniofacial resection (CFR), and endoscopic resection with septectomy (SEP). Specifically, by elucidating the airflow characteristics at sites where crust formation was clinically observed, we aim to investigate whether a canonical airflow environment conducive to crust formation exists regardless of the type of surgery performed.

## Materials and methods

### Patient recruitment and data curation

We conducted a retrospective review of the medical records and CT scans of patients who met the following inclusion criteria: 1) underwent curative endoscopic resection for sinonasal and skull base tumors at Samsung Medical Center, 2) achieved no evidence of disease (NED) status after curative treatment of the tumor and were under follow-up, 3) attended regular outpatient follow-up visits after treatment and presented with persistent crusting in at least one nasal cavity during endoscopic examinations, and 4) had available thin-section CT scan images with a slice thickness of 0. 625mm obtained during the follow-up period.

Patients were excluded from the study if they met any of the following criteria: 1) the surgery performed at Samsung Medical Center was a revision surgery, or 2) the primary tumor site was not controlled after the surgery.

We considered that the tumor’s location would have a greater impact on postoperative nasal airflow than whether it was malignant or benign. Therefore, we categorized and recruited patients based on tumor location. Specifically, we recruited patients who underwent one of three distinct surgical approaches: endoscopic medial maxillectomy (for laterally located tumors, including those involving the medial wall of the maxillary sinus), endoscopic resection with septectomy (for centrally located tumors, including those involving the nasal septum), and endoscopic craniofacial resection (for tumors involving the anterior skull base). These categories ensured the representation of cases involving lateral (e.g., maxillary sinus), central (e.g., posterior septum), and bilateral (e.g., skull base) resections. By including patients across this spectrum, we aimed to examine how the extent of intranasal structure removal affects postoperative nasal airflow and crust formation.

We accessed medical records from April 18, 2023, to February 27, 2024, and collected the patients’ endoscopic findings and CT scans for this study. All data obtained from this review was anonymized for use in our research, and all information was encrypted according to locking protocols, so we had no access to personally identifiable data. Two rhinology specialists reviewed postoperative endoscopic images, and CT scans with a slice thickness of 0. 625 mm or less, taken post-tumor resection to identify and analyze areas of crust formation, considering patients’ symptoms, endoscopic findings, and history of radiotherapy. For patients who received adjuvant radiotherapy, CT scans were typically performed at least one year after the completion of treatment to minimize the effects on the sinus mucosa. This retrospective study, which utilized CT scans obtained during routine clinical care, was conducted with the approval of the Institutional Review Board (IRB) at Samsung Medical Center (IRB No. SMC 2023-03-087-001). The IRB waived the requirement for informed consent due to the retrospective nature of the study. All patient data was anonymized during the research process to ensure confidentiality and prevent any breach of protected health information.

### Generation of three-dimensional postoperative nasal cavity models

Numerical models of the postoperative nasal cavity for subjects were generated using CT scans of the patients with Mimics v23.0 software (Materialise, Leuven, Belgium). Subsequently, the cavity models underwent further processing with Geomagic Wrap 2021 (3D Systems, Rock Hill, SC, USA) to eliminate unrealistic artifacts, such as roughened surfaces generated during the segmentation procedure.

**Fig 1:**
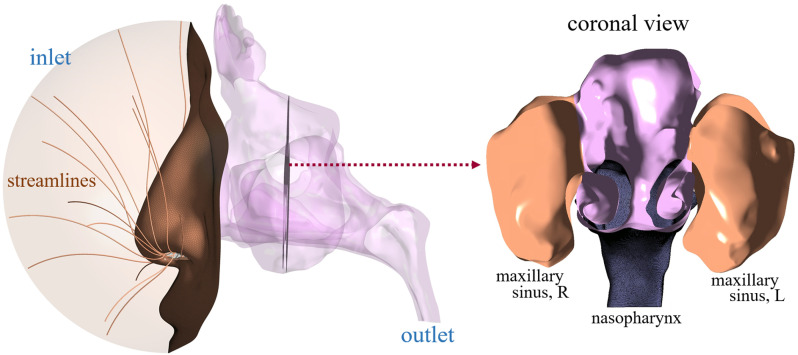
A representative numerical model of a nasal cavity is considered in the present study after undergoing endoscopic resection surgery.

Since the cavity models require only the gross anatomical features, primarily around the nose, to accurately simulate airflow distribution through the nostrils [20,21], a truncated section of the face, with a radius of approximately 5.5–5.6 cm from the nasal valve area, was included in the numerical model as shown in Fig 1. In this study, radical resection was unavoidable in some instances to ensure the complete removal of the tumor, leading to an enlarged airway through the nasal cavity with nasal septum partially or entirely removed as illustrated in Fig 1. Notably, substantial airflow through one or both maxillary sinuses was observed in several subjects. Consequently, the maxillary sinuses were included in the cavity models for all patients. Additionally, frontal, ethmoidal, or sphenoid sinuses were incorporated in specific cases where transit airflow through those sinuses was found non-negligible.

### Numerical methodology for the calculation of parameters

Airflow and air conditioning characteristics in the nasal cavity were analyzed by investigating the air-water vapor mixture’s velocity, pressure, temperature, and mass concentration. Therefore, the continuity, momentum, energy, and species transport equations were solved using ANSYS/Fluent 2023R1 (Canonsburg, PA, USA) under the steady flow assumption. The wall model incorporated in our previous studies [22–25] was utilized to evaluate the distribution of epithelial surface temperature, assuming 100% relative humidity (RH). This computational model effectively eliminates the need for prescribing constant wall temperature values.

The ambient condition of 25 °C and 35% relative humidity (RH) was assumed at the inlet of the domain, and the outlet pressure was adjusted to yield a bilateral airflow rate of 250 mL/s, representing the average airflow rate during calm breathing situations. All variables, except pressure, were extrapolated smoothly at the outlet boundary of the numerical domain from the interior, following the pressure outlet boundary condition specified by ANSYS/Fluent R23.1.

Due to the surgical intervention required for the radical removal of intranasal tumors, the anatomical structures were often significantly deteriorated, leading to a combined nasal cavity without division of the left and right airways. Consequently, the nasal airways in the current cavity models differ significantly from those in non-pathological cases, where viscous friction plays a relatively larger role. Hence, a turbulent flow regime was assumed in this study, and a shear stress transport k-ω turbulence model with low Reynolds correction was employed [7,24,26].

Fluent Meshing 2021R2 was used to generate a combined mesh of seven prism layers placed along the epithelial surface and polyhedral meshes away from the surface. A grid independence study was conducted with 4.1, 6.3, 8.5, and 10.6 million mesh elements for the representative cavity model of the subject (shown in Fig 1), where both frontal and ethmoidal sinuses were included. The study indicates that a mesh system with 8.5 million elements sufficiently resolves the airflow field. Therefore, the same size function was incorporated for the rest of the cavity models, resulting in a range of 7.3–9.9 million mesh elements.

### Comparison of nasal resistance

This study aimed to investigate changes in nasal resistance (NR) by comparing NR between patients who underwent endoscopic tumor resection (surgery group) and a control group of individuals who did not undergo surgery. The control group, consisting of nine subjects, was drawn from data obtained in previous studies [24,25] and represented individuals without surgical intervention.

### Statistical analysis

Statistical analyses were performed using OriginPro 2022b (OriginLab Corporation, Northampton, MA, USA). Descriptive statistics were calculated for all variables, with continuous data presented as mean ± standard deviation. Comparisons between the surgery and control groups were made using two-tailed Student’s t-tests for continuous variables, including NR, air temperature, relative humidity, and surface area-to-volume ratio (SAVR). Statistical significance was set at p < 0.05.

Air conditioning parameters, including air temperature and relative humidity at the choanae, as well as SAVR, were compared between the surgery and control groups to evaluate the impact of radical resection surgeries on the air conditioning capacity of the nasal cavity. The analysis of crust formation sites in relation to wall shear stress (WSS) and wall water-vapor flux (WWF) patterns was conducted qualitatively based on the CFD results and clinical observations.

## Results

**Fig 2:**
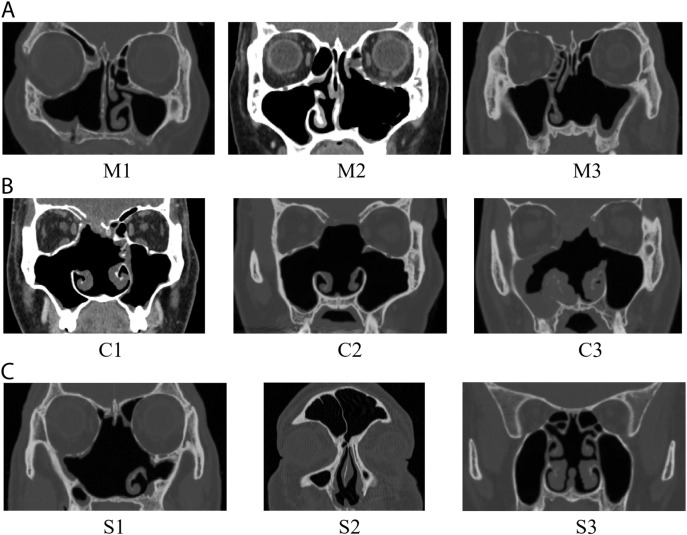
Computed tomography scans of patients in the representative coronal plane: (A) Three subjects (M1–M3) belonging to the endoscopic medial maxillectomy surgery group, (B) Three subjects (C1–C3) belonging to the endoscopic craniofacial resection surgery group, (C) Three subjects (S1–S3) belonging to the endoscopic resection with septectomy surgery group.

A total of nine patients who met the criteria for this study were included and classified into three groups based on the type of endoscopic surgery performed for sinonasal or skull base conditions: endoscopic medial maxillectomy (EMM, N = 3), endoscopic craniofacial resection (CFR, N = 3), and endoscopic resection with septectomy (SEP, N = 3). Detailed descriptions of these procedures, along with corresponding CT images, are shown in Fig 2. The types of surgeries performed, patient demographics, anatomical structures removed, and postoperative sinonasal functional outcomes, measured using the Sino-Nasal Outcome Test (SNOT-22), are summarized in Table 1.

**Table 1:** Summary of patients who underwent resection surgery included in the present study.

Surgery Group	Subject	Age/Sex	Diagnosis	Structures removed	SNOT-22^†^	Date of CT scan	Date of RT^*^ completion
Endoscopic medial maxillectomy	M1	63/F	Nasal cavity cancer (Squamous cell carcinoma)	Right IT^**^, right ethmoid bulla, maxilla medial wall	14	January 2023	August 2019
	M2	64/F	Nasal cavity cancer (Squamous cell carcinoma)	Left IT, left ethmoid bulla, maxilla medial wall	27	March 2023	November 2021
	M3	55/M	Nasal cavity cancer (Adenoid cystic carcinoma)	Left IT, posterior septum, left ethmoid bulla, maxilla medial wall	19	January 2023	March 2020
Endoscopic craniofacial resection	C1	49/M	Olfactory neuroblastoma	Posterior septum, both ethmoid cells, both MT^**^	26	October 2021	N/A
	C2	46/F	Olfactory neuroblastoma	Posterior septum, both ethmoid cells both MT	16	September 2022	February 2021
	C3	42/M	Olfactory neuroblastoma	Posterior septum, both ethmoid cells, both MT	54	September 2022	June 2022
Endoscopic resection with septectomy	S1	53/M	Nasopharyngeal cancer	Posterior septum, both MT, and right IT	41	March 2022	June 2018
	S2	45/M	Pituitary adenoma	Posterior septum	19	June 2021	N/A
	S3	62/F	Chordoma	Posterior septum	N/A	September 2023	N/A

* RT denotes radiation therapy

** IT and MT denote inferior and middle turbinates

† The SNOT-22 scores were assessed for each patient at the time of their postoperative computed tomography scan

For patients who received radiation therapy, the completion date of radiotherapy and the date of the CT scan used in this study are also listed in Table 1. Of the six patients who underwent adjuvant radiotherapy, CT scans taken at least one year after the completion of radiotherapy were used for five. For one patient (subject C3), a CT scan taken three months after radiotherapy was used due to difficulties obtaining a thin-cut CT. A follow-up examination of this patient’s most recent CT scan, performed in March 2024, showed no significant structural changes compared to the CT images used in this study.

### The site of crust formation

Crust formation sites exhibited considerable variability among the patients depending on the diverse locations of primary tumors, encompassing regions from the maxillary sinus to the rostrum, as detailed in Table 2.

**Table 2:** Summary of the locations where crust or bleeding was formed and the nasal resistance.

I. Surgery group			
Surgery type	Subject	Sites of crust/bleeding formation	Bi lateral NR, inlet-choanae (Pa·s/mL)
Endoscopic medial maxillectomy	M1	Inferior surface of the right maxillary sinus	0.0167
	M2	Posterior-medial surface of the left maxillary sinus	0.0223
	M3	Anterior-inferior surface of the right sphenoidal sinus	0.0201
Endoscopic craniofacial resection	C1	Anterior-inferior surface of the left sphenoidal sinus	0.0188
	C2	Anterior surface of the posterior septal stump	0.0316
	C3	Right side of the choanal arch	0.0345
Endoscopic resection with septectomy	S1	Anterior surface of the rostrum	0.0264
	S2	Posterior-inferior surface of the septal perforation	0.0165
	S3	Inferior surfaces of the sphenoidal sinus	0.0219
Mean value of the surgery group (N = 9)			0.0232 ± 0.0064^†^
**II. Control group**			
Control subjects (N = 9)^*^			0.0687 ± 0.0265^†^

* The subjects, representing patients who did not undergo surgery, were cited in our earlier studies [24,25]. Data of the control groups were obtained with an airflow rate of 250 mL/s, consistent with the surgery groups.

† A statistically significant difference was observed between the surgery and control groups (p < 0.001), as assessed by a two-tailed Student’s t-test (Differences are estimated to be statistically significant when the p < 0.05).

Several instances were observed where the ostia leading to the maxillary, ethmoidal, and sphenoidal sinuses were enlarged following resection surgery. This enlargement facilitated increased airflow to these sinuses in the postoperative period. Consequently, this study demonstrated an increased potential for crust formation in areas associated with the paranasal sinuses due to the extensive removal of anatomical structures required for tumor resection. This resulted in a wider affected area compared to the extent typically seen in chronic rhinosinusitis cases undergoing sinus surgery and contrasted with the conditions observed in individuals with normal nasal anatomy. Notably, the data revealed that over 50% of crust formation sites were associated with either the sphenoidal or maxillary sinus (five out of nine cases).

### Airflow characteristics in the region of recurrent crust formation

**Fig 3:**
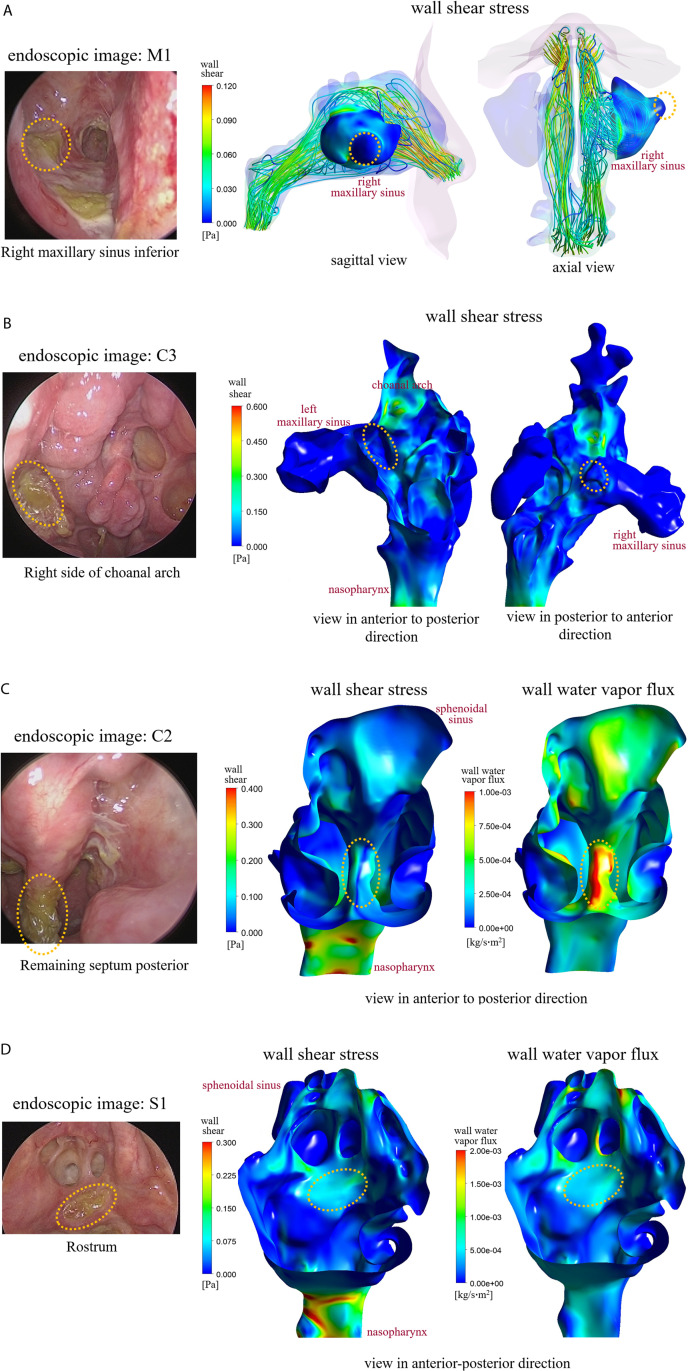
The sites where the crust was clinically observed and their comparison with numerical results. (A) Subject M1, (B) Subject C3, (C) Subject C2, (D) Subject S1.

Fig 3 illustrates two different fluid dynamic environments formed in the regions where crust formation occurs using four representative subjects (M1, C3, C2, and S1). Firstly, in subject M1, as shown in Fig 3A, crust (indicated by the yellow, dotted circle) was observed on the lateral and inferior surfaces of the right maxillary sinus. Examination of the streamlines originating from the right nostril revealed stagnant airflow at the site of crust formation. In other words, the crust formation site is located in the protruding region of the maxillary sinus, where airflow wash-out or ventilation is not very active. Due to the limited air movement, the wall shear stress (WSS) level in this region is significantly suppressed. A similar stagnant airflow behavior was also observed for subject C3 (shown in Fig 3B). In this case, the crust was observed in the region right to the choanal arch, where the airflow was significantly reduced. Again, the site is embedded locally in the protruding airway, resulting in low WSS.

Dissimilar airflow characteristics were observed in the crust sites. For example, Fig 3C illustrates that the crust was formed around the anterior surface of the remaining septum in the posterior cavity in the case of subject C2. Numerical results indicate that this region is characterized by locally increased levels of WSS and wall water-vapor flux (WWF). It is worth noting that the elevated WSS and WWF primarily occur on the surface where the airflow collides with the remaining septum. Similar behavior was also observed for subject S1, as shown in Fig 3D. In this subject, the crust was found in the region of the rostrum where the WSS and WWF were locally increased. Since septectomy was performed for this subject, the cross-sectional area of the airway was significantly increased in the absence of the septum, resulting in an expectedly different airflow pattern from that of the normal subject. Interestingly, higher levels of WSS and WWF were found along the surface of the ostia to the sphenoidal sinus than in the rostrum, but the crust was not formed on that surface.

**Fig 4:**
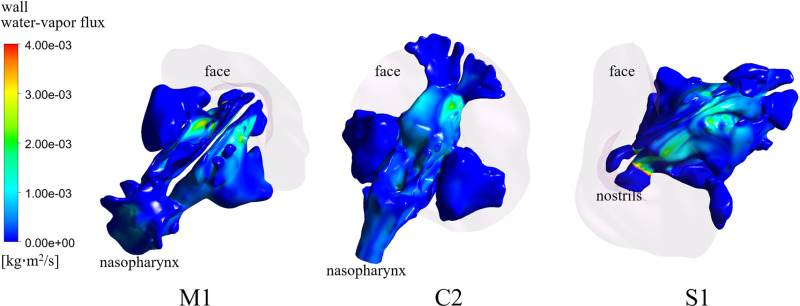
Wall water-vapor flux distributions along the nasal roof for the subjects M1, C2, and S1.

Fig 4 shows that WWF distribution mainly along the nasal roof of the nasal cavities for three representative subjects (M1, C2, and S1) from the EMM, CFR, and SEP groups, respectively. These three subjects were also discussed in Fig 3. It was observed that a jet-like airflow originating from the nasal valve area was directed toward to the superior surface of the nasal cavity in these subjects, leading to significantly increased WWF. Streamline patterns resulting from a jet-like airflow directed towards the nasal roof were visualized in the particle traces for subjects M1 and C2. These patterns were compared with those of a representative normal subject whose nasal anatomy was unaffected by surgery, as reported in our previous studies [22–24]. The visualizations for subjects M1, C2, and the representative normal subject are provided in S1–S3 Particle Trace Recordings, respectively, included in the Supplementary Files. For the same reason, WSS also increased at the same locations. However, as explained in Fig 3, crusting was not observed in those areas.

### Nasal resistance and air conditioning parameters

NR is summarized in Table 2. Bilateral NR shows variations between the resection surgery groups, with the CFR group exhibiting the highest mean value. The largest standard deviation was also observed in the CFR group, primarily due to the low value of subject C1. The four highest NR values were noted in subjects C3, C2, S1, and M2 (0.0345, 0.0316, 0.264, and 0.0223 Pa·s/mL). For comparison, the NR of the group that underwent endoscopic tumor resection was compared with data from nine control subjects without any history of nasal surgery, as reported in our previously published studies [24,25].

The group that underwent endoscopic tumor resection, referred to as the surgery group in Table 2, exhibited lower NR compared to the control group (0.0232 ± 0.0064 vs 0.0687 ± 0.0265 Pa·s/mL, p < 0.001), suggesting that the surgery compromised the ability to maintain optimal resistance for nasal homeostasis.

Air temperature and relative humidity evaluated at the choanae, and surface area-to-volume ratio (SAVR) are summarized in Table 3. While the air temperatures did not differ significantly among the three resection surgery groups, relative humidity exhibited some variations; the CFR group showed the lowest relative humidity (83.8 ± 2.3%). Notably, SAVR had the lowest value of 0.279 ± 0.015 mm^-1^ in the CFR group. The endoscopic resection surgery group showed a much lower relative humidity level compared to the control group (85.0 ± 2.1, 92.9 ± 3.2%, p < 0.001), given the same inspiratory airflow rate. The SAVR shows that radical resection surgeries such as maxillectomy, CFR, and septectomy resulted in a much lower SAVR (0.354 ± 0.135 mm^-1^) than the control groups (1.031 ± 0.100 mm^-1^) with p < 0.001.

**Table 3:** Air-conditioning-related variables at the choana and the surface area-to-volume ratio for the nasal cavity were evaluated for the surgery and control groups.

I. Surgery Group			
Surgery type	Air temperature, choana (^o^C)	Relative humidity, choana (%)	Surface area-to-volume ratio^‡^ (mm^-1^)
Endoscopic medial maxillectomy	31.4 ± 0.1	85.7 ± 0.5	0.331 ± 0.069
Endoscopic craniofacial resection	31.3 ± 0.2	83.8 ± 2.3	0.279 ± 0.015
Endoscopic resection with septectomy	31.4 ± 0.2	85.6 ± 3.1	0.454 ± 0.208
Mean value of the surgery group (N = 9)	31.4 ± 0.2^†^	85.0 ± 2.1^†^	0.354 ± 0.135^†^
**II. Control Group**			
Control subjects (N = 9)^*^	32.3 ± 0.5^†^	92.9 ± 3.2^†^	1.031 ± 0.100^†^

* The subjects, representing patients who did not undergo surgery, were cited in our earlier studies [24,25]. Data from the control groups were obtained with an airflow rate of 250 mL/s, consistent with the surgery groups

† Statistically significant differences were observed between the surgery and control groups for air temperature (p < 0.001), relative humidity (p < 0.001), and surface area-to-volume ratio (p < 0.001), as assessed by a two-tailed Student’s t-test (Differences are estimated to be statistically significant when the p < 0.05)

‡ The surface area-to-volume ratio was calculated based on the airflow volume through which the airflow passes

## Discussion

This CFD investigation into the effects of radical nasal surgeries on one of the most common postoperative sinonasal morbidities, crust formation, has yielded several noteworthy findings. Notably, crust formation does not necessarily occur solely in regions exhibiting high WSS or WWF; it can also manifest in geometrically secluded areas where air-washout is significantly suppressed. This observation indicates that crust formation may be influenced by at least two distinct fluid dynamic environments, underscoring the challenge of accurately predicting surgical and nasal functional outcomes.

Radical endoscopic resection surgeries, which significantly alter nasal morphology, may give rise to various complaints. Specifically, sinonasal morbidity following endoscopic endonasal skull base surgery can dramatically impact patient quality of life. A study by Awad et al. [17] reported that the most prevalent symptoms of sinonasal morbidity after endoscopic skull base surgery include nasal crusting (50.8%), nasal discharge (40.4%), nasal airflow blockage (40.1%), followed by disturbances in olfaction (26.7%). In this context, our investigation, directly comparing clinical observations via endoscopic examination with numerically simulated results, is expected to enhance understanding of crust formation etiology.

Extensive removal of intranasal structures may disrupt the distribution function of the turbinates. Several studies have suggested less morbidity if the middle turbinate can be preserved [6,17]. However, the precise role of the middle turbinate in airflow distribution remains unclear, with contradictory observations reported in the literature [14] necessitating further investigation into their mechanisms and clinical implications. Both middle turbinates were removed in all three subjects of the CFR group considered in this study. Surgery effectively eliminated structural barriers deflecting the airstream from the nasal valve into the inferior and middle meatuses. This airflow pattern induced locally elevated levels of WSS and WWF along the nasal roof, as illustrated in Fig 4. However, clinical observation revealed that crust did not form along the nasal roof (as shown in Fig 3C), suggesting that the magnitude of WSS (or WWF) alone may not be a sufficient condition for crust formation. Another conjecture is that different parts of the epithelial surface of the nasal cavity may exhibit varying resistance to mechanical stimuli, leading to regional differences in the threshold value of WSS (or WWF) during crust formation. A larger number of samples would be necessary to test these hypotheses in the future. Loss of the olfaction function was not examined in this study, even though the airflow near the superior meatus was significantly disturbed (Fig 4). However, there is a report that anterior skull base surgery would appear to be associated with an increased rate of anosmia post-operatively [10].

The study observed a considerable reduction in NR after the removal of many intranasal structures (0.0232 ± 0.0064 Pa·s/mL) compared to non-surgery controls (0.0687 ± 0.0265 Pa·s/mL), as shown in Table 2. This reduction is attributed to increased nasal airflow due to surgery, leading to decreased airflow through narrower, normal meatuses. Additionally, the air conditioning capacity of the nasal cavity can be assessed by the thermodynamic state of the air at the nasopharynx. The RH at the choanae shows some variation after radical tumor surgery, as shown in Table 3 (83.8–85.7%). The CFR group, exhibiting the lowest RH (83.8 ± 2.3%), also has the lowest SAVR (0.279 ± 0.015 mm^-1^). Although statistically meaningful conclusions are impossible due to the limited number of samples (N = 3), this study suggests that the CFR group may be more prone to nasal dryness after radical surgery.

Physiological variables of the present study can also offer insights into the possibility of developing severe complications from the perspective of the ENS. Several earlier clinical studies have reported mild sinonasal complaints after CFR [9,10]. A numerical study involving three patients who underwent craniofacial resection (CFR) to treat extensive anterior skull base lesions also reported the absence of empty nose syndrome (ENS) symptoms post-surgery [7]. However, after reviewing medical records by two laryngologists, ENS was developed in four out of nine subjects considered in this study (subjects M2, C2, C3, and S1). Notably, three subjects (C3, S1, M2) exhibited the three highest SNOT-22 scores in order (Table 1), and these subjects had the 1st, 3rd, and 4th highest NR (Table 2). Although subject C2 did not have a relatively high SNOT-22 score, it exhibited the 2nd highest NR. Furthermore, subject S1 experienced the lowest relative humidity of 82.0% at the nasopharynx, suggesting a potential correlation between nasal dryness and reduced relative humidity for this subject.

In the presence of substantial anatomical variability, the additional anatomical modifications resulting from tumor removal surgeries highlight the importance of personalized approaches [6]. For instance, while locally increased WSS and WWF occur in similar locations on the nasal roof due to the collision of a jet-like airstream with the superior surface for subjects M1 and C2 (Fig 4), crust formation developed in entirely different epithelial surfaces of the cavity (Fig 3A and 3C). These findings underscore the importance of considering individual anatomical variations and surgical nuances in understanding and addressing complications associated with sinus cancer surgeries. Future studies could explore predictive models based on individual anatomical variations to enhance surgical planning. Prospective studies tracking patients over extended periods could provide insights into the long-term implications of altered nasal airflow dynamics post-surgery because the quality-of-life measures following endoscopic skull base surgery using the Anterior Skull Base questionnaire and the SNOT-22 questionnaire showed significantly improved nasal scores at six months compared with immediately after surgery [9].

Our CFD results indicated that relative humidity in the nasopharynx substantially decreased (Table 3), suggesting a deterioration of the nasal cavity’s overall humidification function following resection surgery. This reduction in humidity increases nasal dryness, creating a more vulnerable environment for crust formation. Additionally, postoperative deterioration of the nasal cavity’s warming function, reflected in decreased air temperature in the nasopharynx (Table 3), may also contribute to crust formation. Changes in temperature-dependent parameters, such as increased mucus viscosity, altered mucociliary movement, and reduced saturation water vapor, could influence this process. However, due to the lack of detailed studies on temperature variation in nasal airflow, any quantitative assessment of the warming function’s impact on crust formation must be approached cautiously. Future investigations are necessary to address these gaps.

The present study has several limitations that must be cautiously interpreted. Firstly, the number of samples is limited to N = 9, compromising the accuracy of the statistical values. Although this study included more samples than several existing numerical studies dealing with the effect of radical surgery on airflow characteristics [6,7,12,19,27], the reliability of the several conjectures describing airflow characteristics in the crust site should be increased in future research. Additionally, this study did not intend to assess differences between the three surgical groups (i.e., EMM, CFR, and SEP groups) regarding physiological variables with the limited number of samples. Moreover, the results of the ENS6Q questionnaires were unavailable; therefore, two laryngologists were able to determine ENS patients by reviewing the medical records of all nine patients. Thus, the severity of symptoms was based on the SNOT-22 score, and the discussion of ENS symptoms should be cautiously interpreted. Third, we acknowledge that we were unable to fully control various factors that could influence nasal airflow changes following tumor resection. Several factors, including a history of radiation therapy, postoperative nasal irrigation, and smoking, may affect mucociliary function, with radiation therapy being a well-established cause of mucociliary dysfunction. We carefully considered the timing of CT scan acquisition to minimize the potential impact of radiation-induced mucosal changes in our study. The CT scans used for our CFD analysis were obtained between 5 and 50 months after the completion of radiation therapy. Although most of the scans were collected several months post-treatment, we did not specifically evaluate whether mucociliary clearance had fully recovered. Therefore, while structural changes resulting from surgical resection primarily influenced nasal airflow patterns, we cannot exclude the possibility that radiation-induced mucociliary dysfunction also contributed to these changes. Future studies should aim to delineate the distinct effects of radiation therapy from those of surgical alterations on nasal physiology and associated morbidities, such as crust formation. Moreover, smoking and nasal irrigation are both known to impact mucosal healing. Smoking has been widely reported to impair ciliary function, delay wound healing, and increase postoperative crust formation, which may have introduced variability in patient outcomes. However, due to the retrospective nature of this study, we were unable to obtain detailed smoking histories for our cohort.

Additionally, the latent heat transfer mechanism arising from water-vapor evaporation was not considered in this study, based on prior observations that latent heat plays a minimal role in patient-reported effects [25]. Nevertheless, future studies should further investigate the effect of latent heat transfer on crust formation to provide a more comprehensive understanding.

## Conclusion

The present study demonstrated that recurrent crust formation occurs in specific areas of the nasal cavity following nasal tumor surgery, especially in regions with deteriorated anatomy. Our findings suggest the involvement of two distinct fluid dynamic environments in crust formation: localized increases in WSS or stagnant flow dynamics. This study represents the first attempt to establish a relationship between crust formation and airflow dynamics, offering insights into predicting delayed mucosal recovery.

Additionally, the study observed a substantial decrease in relative humidity in the nasopharynx among subjects who underwent resection surgery, attributed to reduced SAVR. This decrease indicates impaired humidification, emphasizing the importance of continuous nasal humidification for patients with nasal tumors after endoscopic resection. These findings provide scientific evidence supporting the necessity of maintaining adequate nasal moisture during post-surgical care.

## Supporting information

S1 Particle Trace Recording 01_*particle_trace_EMM_M1_FHD.mp4*. Particle trace in MPEG-4 format with Full High Definition (FHD) resolution shows weightless particles passively moving along the streamlines of the steady nasal airflow for subject M1 from the endoscopic medial maxillary group. This animation was recorded at a speed of 512 frames per second using the Sweep Animation option in CFD-Post 2023R1.
(MP4)

S2 Particle Trace Recording 02_*particle_trace_CFR_C2_FHD.mp4*. Particle trace in MPEG-4 format with FHD resolution shows weightless particles passively moving along the streamlines of the steady nasal airflow for subject C2 from the endoscopic craniofacial resection group. This animation was recorded at a speed of 512 frames per second using the Sweep Animation option in CFD-Post 2023R1.
(MP4)

S3 Particle Trace Recording 03_*particle_trace_NORMAL_FHD.mp4*. Particle trace in MPEG-4 format with FHD resolution shows weightless particles passively moving along the streamlines of the steady nasal airflow for a representative subject N1 from the control group. This animation was recorded at a speed of 512 frames per second using the Sweep Animation option in CFD-Post 2023R1.
(MP4)
